# Factors associated with self-reported diagnosis of depression in Kenya: insights from 2022 Demographic and Health Survey

**DOI:** 10.1186/s12889-025-24304-9

**Published:** 2025-08-29

**Authors:** Evans Omondi, Wingston Felix Ng’ambi, Bylhah Mugotitsa, Chance Ng’ambi, Miriam Sitienei, Steve Cygu, Collins Odhiambo, Samuel Iddi, Damazo T. Kadengye, Agnes Kiragga

**Affiliations:** 1 African Population and Health Research Center, Nairobi, Kenya; 2 Institute of Mathematical Sciences, Strathmore University, Nairobi, Kenya; 3 Health Economics and Policy Unit, Department of Health Systems and Policy, Kamuzu University of Health Sciences, Lilongwe, Malawi; 4 Strathmore University Business School, Strathmore University, Nairobi, Kenya; 5 Data Science and Statistical Consulting Centre, Lilongwe, Malawi; 6 African Institute for Mathematical Sciences, Limbe, Cameroon; 7 Department of Pediatrics, University of Illinois College of Medicine-Peoria, Peoria, 61604 IL USA; 8 Department of Statistics and Actuarial Science, University of Ghana, Legon-Accra, Ghana

**Keywords:** Depression, Stress, Mental disorder, Anxiety, Psychology, Mental well-being

## Abstract

**Background:**

Mental health issues, particularly depression, have seen a significant increase worldwide in recent years, raising global concern. Depression is projected by the World Health Organization to become the leading cause of mental illness by 2030. This condition severely impacts the quality of life and psychosocial functioning of those affected, underscoring the need for effective interventions and awareness. The 2022 Kenya Demographic and Health Survey is the first Demographic and Health Surveys in Africa to incorporate the mental health diagnosis module. Therefore, this study assesses socio-economic, demographic characteristics and health conditions associated with depression in Kenya.

**Methods:**

This secondary analysis uses data from the 2022 Kenya Demographic and Health Survey, in which participants were selected from households within designated demographic surveillance areas across Kenya. A two-stage cluster sampling method was applied to achieve national representativeness. The study examined associations between socio-demographic characteristics that included age, gender, education level, marital status, household wealth index, and urban/rural residence, as well as chronic diseases such as heart and lung diseases, hypertension and diabetes, and self-reported depression using logistic regression analysis. Both crude and adjusted odds ratios (ORs) for self-reported depression were assessed, with statistical significance set at a *p*-value of $<0.05$. Additionally, spatial analyses of self-reported depression prevalence were conducted to identify regional patterns.

**Results:**

A total of 31354 men and women were assessed. The prevalence of self-reported depression was 2.6%. Being a middle-income earner was associated with increased likelihood of depression (aOR:1.12, 95% CI: 1.03-1.22). Being hypertensive, diabetic, and having heart and lung diseases increased the likelihood of having depression by at least two-fold, (aOR:3.71, 95% CI: 3.49-3.95, aOR:1.14, 95% CI: 0.96-1.35, aOR:3.10, 95% CI: 2.68-3.57 and aOR:3.21, 95% CI: 2.84-3.62, respectively).

**Conclusion:**

The proportion of participants who self-reported depression was low. Besides, there was a considerable variation in self-reported depression across different counties in Kenya. Furthermore, there was variability in depression among different socio-demographic groups. Therefore, there is a need to target mental health interventions to persons with chronic conditions and those in middle economic class as they are greatly affected with depression.

## Introduction

Depression has emerged as a major contributor to global mental health distress and dysfunction. Characterized as a medical condition, depression influences, cognition, and behaviour [1]. The etiology of depression encompasses a complex interaction among dysfunctional mood regulation mechanisms within the brain, genetic predisposition, and exposure to adverse life events [2]. In the Kenyan context, depression represents a significant source of morbidity [3, 4]. According to Globally-Minded [5], Kenya has an approximately 1.9 million cases of depression ranking 5th after South Africa whose cases are reported to be 2.4 million, the Democratic Republic of Congo with 2.9 million cases, Ethiopia with 4.5 million cases and Nigeria with 7.1 million cases. According to the Kenya National Commission on Human Rights, approximately 25% and 40% of the outpatients (individuals who visit health facilities for consultations or treatment without being admitted overnight) and inpatients (individuals who are admitted and stay in health facilities for continued care) suffer from mental illness of which the majority of the diagnoses are depression, stress, and anxiety disorders [6]. These statistics point to the imperative need for the assessment of depressive symptoms and the identification of correlating factors within the Kenyan population. Depression is a major public health concern in Kenya, driven by high prevalence rates, socioeconomic challenges, limited mental health resources, and stigma. Understanding the demographic and socio-economic factors linked to depression is crucial for designing effective interventions [7, 8]. Without proper care, mental health conditions can worsen physical health, lower productivity, and strain the healthcare system. Addressing depression is therefore a national priority that calls for comprehensive and targeted strategies.

Many studies on depression have focused on describing incidence rates, examining trends over time, and identifying factors linked to depression and anxiety [9], all with the goal of reducing the prevalence of these and related mental health disorders [10]. Notably, in the Kenyan demographic, poverty and unemployment have been pinpointed as salient precipitants of depression [11]. Seth et al. [12] highlighted in their study that the Human Immunodeficiency Virus (HIV) is a critical determinant of mental health challenges, noting that individuals diagnosed with HIV exhibit high incidences of mental health disorders, including but not limited to depression, stress, anxiety, and trauma. Moreover, Abas et al. [13] documented that depressive manifestations and psychological distress among persons living with HIV correlates with sub-optimal self-assessments of physical health and diminished adherence to Antiretroviral Therapy, consequentially elevating mortality risks. According to Kumar et al. [14], substance misuse, including alcohol and drug use, is associated with an increased prevalence of depression and broader mental health challenges.

Research by Kuria et al. [15] provided a high prevalence of depression amongst individuals with alcohol dependency, estimated at 63.8%. Additional factors contributing to high prevalence of depression include stress-inducing life events such as bereavement or dissolution of relationships, personality dispositions characterized by low self-esteem or excessive self-criticism, genetic predispositions evidenced by familial histories of severe depression, life transitions including pregnancy and childbirth, menopause, experiences of isolation, and chronic illnesses [16]. Moreover, media consumption has emerged as a significant factor influencing mental health outcomes thereby warranting its inclusion in studies exploring determinants of depression. Previous research has shown that frequent exposure to media, that include social media, can have both positive and negative effects on psychological well-being [17, 18]. On one hand, media platforms can serve as crucial sources of information, raising awareness about mental health issues and potentially reducing stigma around seeking help [19]. On the other hand, excessive media use, particularly passive consumption such as watching television or engaging with social media, has been linked to heightened anxiety, depressive symptoms, and reduced quality of sleep due to exposure to distressing or overwhelming content [20, 21].

Prior to the dissemination of the 2022 Kenya Demographic Health Survey (KDHS), a notable gap existed in the national dataset concerning depression in Kenyan. With the emergence of this dataset, there is an obvious need for a comprehensive analysis to delve into understanding the patterns of depression across the country. This in-depth analysis is crucial for several reasons. First, this study bridges the informational gap, enlightening the breadth and depth of mental health conditions faced by the citizenry. Second, this investigation is instrumental in pinpointing demographic segments inherently more susceptible to depression and mental health challenges. The understanding derived from this research is a foundation for developing targeted interventions, thereby optimizing resource deployment to meet the individual needs of a specific vulnerable cohorts. In essence, an exploration into the dynamics of depression in Kenya is indispensable for the enhancement of communal health welfare. Thus, a study of depression in Kenya is of practical necessity for promoting the overall well-being of the population. This can help advocate for a holistic approach to health. In championing for an integrated mental health paradigm, insights gained from this study supports the establishment of a health infrastructure that promotes the overall well-being of the population thereby contributing substantively towards the realization of a healthy citizenry for economic development.

## Methods

### Data source

This study is a secondary analysis of data from the 2022 Kenya Demographic and Health Survey (KDHS), a part of the broader Demographic and Health Surveys (DHS) program conducted across low and middle-income countries approximately every five years [22]. These surveys, led by the National Bureau of Statistics in collaboration with Inner City Fund (ICF) International and other development partners, use a cross-sectional design to assess health indicators, including mental health conditions like depression and anxiety. These assessments are done through self-reporting where the information is provided directly by individuals about themselves and the participants answer questions based on their personal perceptions, experiences, or behaviors.

The KDHS employs a two-stage cluster sampling approach to ensure national representativeness. In the first stage, a stratified random sample of enumeration areas (EAs), often based on recent census data, is selected with a probability proportional to their population size. Each EA serves as a cluster, containing a group of nearby households. Within each stratum, a predetermined number of EAs is independently selected, with the likelihood of selection directly tied to the EA’s size. In the second stage, a systematic random sample of households is chosen from the listed households within the selected EAs. Data on individuals aged 15–49 are then gathered through household questionnaires, with one individual per household invited to participate in the mental health module, which covers depression and anxiety.

For the 2022 KDHS, the survey initially targeted a sample size of 42,300 households to provide reliable estimates at the national and regional levels, with distinctions between urban and rural populations. The survey was conducted in all 47 counties in Kenya, encompassing various demographics to capture both physical and mental health indicators. The response rate for KDHS 2022 was approximately 95%, ensuring comprehensive data collection and high representativeness of the target population.

The KDHS mental health assessment is carried out using the DHS depression and anxiety module. In this module, respondents are asked about their medical history, including any diagnosis of depression or anxiety by a healthcare professional, as well as current treatment status for these conditions [22, 23]. The DHS module captured binary responses (yes/no) on prior diagnosis and current treatment status for mental health conditions [24]. Associated with this there were thirteen questions about chronic diseases such as hypertension, diabetes, lung disease, and heart diseases which were also captured as binary responses (yes/no) [24]. This structured module facilitates accurate cross-national comparisons. For broader demographic characteristics, KDHS categorized data by education level, household wealth index, age, marital status, occupation, residence type (urban/rural), and ethnicity, using multi-level categories to enable detailed analysis across these variables. The wealth index was constructed using household asset data such as ownership of durable goods, access to clean water, and electricity, which was then compiled into wealth quintiles. This stratification supports socioeconomic analysis across different population groups in Kenya and how socioeconomic factors influence health outcomes, including mental health.

The data collection was conducted by trained enumerators from the Kenya National Bureau of Statistics (KNBS), who specialized in survey methodology and health data collection. This intensive data-gathering process spanned six months, beginning in February 2022 and concluding in July 2022 and ensured high standards of reliability and accuracy. To address missing data, KDHS used multiple imputation techniques, where missing values in key variables were estimated based on observed relationships, thus, reducing potential biases in the results. Additionally, sampling weights were applied to correct for differences in selection probability and non-response so as to ensure that the findings were representative of the national population. These weights were further adjusted based on population benchmarks such as age, gender, and geographic distribution from the 2019 population and housing census data. For cases with substantial missing data that could affect the reliability of mental health estimates, imputations were guided by auxiliary data, minimizing bias and enhancing the accuracy of depression and anxiety metrics.

### Ethical compliance and participant consent

The study uses secondary data officially published by the Kenya National Bureau of Statistics (KNBS) and Inner City Fund (ICF) International - The DHS Program. The data provided by these institutions are already anonymized, meaning that personal identifiers have been removed to protect the privacy of individuals. The use of data from KNBS is governed by the Statistics Act of Kenya 2006, which designates KNBS as the official custodian of statistical data in the country. This Act gives KNBS the responsibility to collect, compile, analyze, and disseminate statistical information for public use, including research. Researchers utilizing data from KNBS are working within this Act’s framework, ensuring that the data are handled responsibly and that their use contributes to the public good.

### Variables and statistical analysis

The independent variables considered for this study were age (in years), gender, residence, educational level, religion, ethnicity, media exposure, wealth index, hypertension, diabetes, heart disease, lung disease, and occupation. All categorical variables were presented as frequencies and percentages. To evaluate the relationship between depression and categorical variables, Fisher’s exact and chi-squared tests were applied. Odds ratios and their corresponding 95% confidence intervals (CIs) were utilized as indicators of association. Additionally, a heat map is constructed to illustrate the distribution of self-reported depression across Kenya. Each county’s prevalence of self-reported depression was calculated and mapped using Geographic Information System (GIS) shape files that outline Kenya’s administrative divisions. This approach provides a visual representation of regional disparities in depression prevalence, aiding in the identification of high-burden areas for targeted interventions.

Logistic regression, or the logit model, is a frequently employed statistical model in the realms of classification and predictive analytics. This model is designed to estimate the likelihood of an event happening, taking into account various independent variables within a provided dataset. In health care, this model is commonly used to predict the likelihood of a disease or illness occurrence in each population. Thus, they can set preventive measures for individuals showing a higher propensity for specific illness. A survey-weighted logistic regression is employed to account for the complex sampling design and to appropriately account for weighting. The data were extracted and initially stored in STATA before being transferred to R. All statistical analyses were conducted using Stata (V.18, Stata Corp, College Station, Texas, USA) and R (version 4.4.1, R Core Team) and R Studio (version 2024.09.0+375, R Studio Team) software.

## Results

### Characteristics of the sample

The sample consisted of individuals aged 15-49 years, with a balanced representation across age groups, gender, and various socio-demographic characteristics. Table 1 provides detailed characteristics of the sample, highlighting key demographic factors, including sex, age, residence, education level, religious affiliation, ethnicity, media exposure, household wealth index, existence of chronic diseases, and occupation. The results show that 53.9% of the participants were female and 46.1% were male. In terms of age, the distribution across different age groups ranges from 21.3% of the individuals aged 15-19 years to 2.5% of the individuals aged 50-54 years. The largest age group is 15-19, while the smallest is 50-54, highlighting a youthful overall sample. There are more participants from rural areas (62.5%) than urban areas (37.5%). This suggests a higher representation of rural residents in the study. Furthermore, the findings includes 9.3% of the individuals with no education, 37.2% with primary education, 37.3% with secondary education, and 16.2% with higher education. The most common religious affiliation is Protestant at 34.3%, followed by Catholic at 18.8% and Evangelical at 18.6%. Less common affiliations include Orthodox (0.2%) and Hindu (0.1%), while 2.5% of the participants reported having no religion.

The results also show that the largest ethnic groups in the study are the Kalenjin (17.0%), Kikuyu (13.9%), and Luhya (13.0%). The smallest ethnic groups include the Embu (1.5%) and Taita (1.2%). In relations to media exposure, most individuals (89.2%) read newspapers or magazines less than once a week, and 37.8% listen to the radio less frequently. Television viewership is evenly split, with 49.2% watching less than once a week and 50.8% at least once weekly. Internet usage is also skewed, with 62.9% of the individuals using it less than once a week. The wealth distribution is fairly even across the quintiles, with the richest quintile comprising 17.1% of the individuals and the poorest quintile accounting for 21.6% of the individuals. In addition, majority of the participants do not have hypertension (94.2%), diabetes (99.1%), heart disease (99.1%), or lung disease (98.9%). Finally, in terms of occupation, the largest group is “Not working” (35.5%), followed by “Agriculture - employee” (18.6%). Smaller groups of individuals include those in clerical jobs (0.9%) and agriculture - self-employed (0.9%). These characteristics provide a representative cross-section of the Kenyan population in urban and rural settings, forming the basis for examining the associations between these socio-demographic factors and self-reported mental health outcomes.

### Distribution of self-reported depression

Table 1 summarizes the features associated with depression. Overall, 97.4% (30,530 individuals) reported that a healthcare professional did not diagnose them with depression or anxiety, while a small proportion, 2.6% (824 individuals), did report depression or anxiety. Age appears to be a significant factor, with the incidence of reported depression varying notably across age groups. The age group 15-19 years old reported depression at a rate of 0.875%, while those aged 50-54 reported it at a rate of 4.19%. The highest percentage of reported depression was in the 40-44 age group, at 4.73%. Notably, the proportion of depression increases with age up until the 40-44 age bracket and then declines with further increases in age. The differences across age groups are statistically significant. Gender also influences the reported rates of depression, with females showing a significantly higher proportion (2.85%) compared to males (2.21%). A majority of individuals reside in rural areas, with a 2.69% reporting depression. Urban residents account for 2.35% of those who reported depression.

**Table 1:** The distribution of factors associated with self-reported depression among people aged 15-54 years in Kenya

Characteristics	Has depression				Total		*P*-value
	No		Yes				
	n	%	n	%	n	%	
Total	30530	97.4	824	2.6	31354	100	
Sex							
Male	14110	97.8	343	2.21	14453	46.1	<0.001
Female	16420	97.1	481	2.85	16901	53.9	
Age							
15-19	6629	99.1	59	0.875	6688	21.3	<0.001
20-24	5309	98.5	85	1.46	5394	17.2	
25-29	4808	97.6	134	2.37	4942	15.8	
30-34	4013	97	135	3.05	4148	13.2	
35-39	3771	96.1	146	3.93	3917	12.5	
40-44	2892	95.3	131	4.73	3023	9.6	
45-49	2359	96.5	99	3.54	2458	7.8	
50-54	749	95.8	35	4.19	784	2.5	
Residence							
Urban	11409	97.7	340	2.35	11749	37.5	<0.001
Rural	19121	97.3	484	2.69	19605	62.5	
Education level							
No education	2858	97.5	54	2.47	2912	9.3	<0.001
Primary	11318	97	352	2.98	11670	37.2	
Secondary	11433	98	269	1.99	11702	37.3	
Higher education	4921	97.1	149	2.93	5070	16.2	
Religion							
Catholic	5748	97.2	158	2.77	5906	18.8	<0.001
Protestant	10474	97.6	288	2.4	10762	34.3	
Evangelical	5680	97.5	156	2.55	5836	18.6	
African Instituted Churches	2210	97.7	52	2.33	2262	7.2	
Orthodox	53	96.6	1	3.4	54	0.2	
Islam	4777	97.6	97	2.35	4874	15.5	
Hindu	30	98.1	1	1.93	31	0.1	
Tradition	118	98.6	1	1.35	119	0.4	
No religion	737	95.3	51	4.72	788	2.5	
Other	703	97.9	19	2.06	722	2.3	
Ethnicity							
Embu	453	99.2	8	0.831	461	1.5	<0.001
Kalenjin	5158	97	168	2.99	5326	17.0	
Kamba	2714	98.2	38	1.77	2752	8.8	
Kikuyu	4242	97.7	123	2.33	4365	13.9	
Kisii	1705	97.3	53	2.71	1758	5.6	
Luhya	3947	96.9	138	3.06	4085	13.0	
Luo	3852	97.6	97	2.41	3949	12.6	
Maasai	623	95.4	35	4.63	658	2.1	
Meru	1715	96.5	55	3.49	1770	5.6	
Mijikenda	1602	97.9	39	2.07	1641	5.2	
Somali	2311	98.4	22	1.61	2333	7.4	
Taita	372	97.8	10	2.24	382	1.2	
Other	1836	98.6	38	1.44	1874	6.0	
Media exposure							
Newspaper/magazine							
Less than one day a week	27231	97.4	741	2.56	27972	89.2	0.521
At least one day a week	3299	97.5	83	2.53	3382	10.8	
Radio							
Less than one day a week	11534	97.1	329	2.93	11863	37.8	0.281
At least one day a week	18996	97.6	495	2.36	19491	62.2	
Television							
Less than one day a week	14977	96.9	436	3.05	15413	49.2	0.021
At least one day a week	15553	97.8	388	2.18	15941	50.8	
Internet							
Less than one day a week	19189	97.3	533	2.75	19722	62.9	0.091
At least one day a week	11341	97.7	291	2.29	11632	37.1	
Wealth index							
Poorest	6645	97.4	143	2.63	6788	21.6	<0.001
Poorer	5642	97.3	161	2.66	5803	18.5	
Middle	6166	97.2	187	2.76	6353	20.3	
Richer	6875	97.5	187	2.53	7062	22.5	
Richest	5202	97.7	146	2.26	5348	17.1	
Has hypertension							
No	28906	97.9	645	2.12	29551	94.2	<0.001
Yes	1624	90.9	179	9.08	1803	5.8	
Has diabetes							
No	30282	97.5	800	2.52	31082	99.1	<0.001
Yes	248	94	24	6.01	272	0.9	
Has heart disease							
No	30291	97.5	790	2.49	31081	99.1	<0.001
Yes	239	91.1	34	8.88	273	0.9	
Has lung disease							
No	30220	97.5	789	2.47	31009	98.9	<0.001
Yes	310	90.4	35	9.63	345	1.1	
Occupation							
Not working	10989	98.6	157	1.41	11146	35.5	<0.001
Professional/Technical/Managerial	4077	97	140	2.97	4217	13.5	
Clerical	278	97.6	10	2.42	288	0.9	
Sales	1600	97.5	45	2.5	1645	5.3	
Agriculture - self employed	289	97.4	7	2.56	296	0.9	
Agriculture - employee	5615	96.4	205	3.62	5820	18.6	
Household and domestic	1342	96.6	60	3.4	1402	4.5	
Services	1294	97.2	46	2.83	1340	4.3	
Skilled manual	2611	97.1	82	2.89	2693	8.6	
Unskilled manual	2435	97.1	72	2.95	2507	8.0	

Education level shows a association with reported depression. From these findings, individuals with higher education report depression at a rate of 2.93%, compared to 2.47% among those with no education. This trend suggests that higher education levels correlate with a higher reported depression, and the differences in reported depression across education levels are significant. Furthermore, the results suggest that a extra level of education leads to a higher tendency of reporting any symptoms related to depression or anxiety. Religion also shows varied rates of reported depression. For instance, those with no religion report the highest rate of depression (4.72%), while Hindus report the least (1.93%). The impact of religious affiliation on reported depression is statistically significant Regarding ethnicity, Maasais report the highest rate of depression (4.63%), followed by Meru at (3.49%) while the Embu ethnicity reports the lowest (0.831%). Significant differences in reported depression across ethnic groups are indicated.

The results also provide information on the consumption of various media types and the socioeconomic status of a population, juxtaposed with the incidence of reporting for depression. While magazine reading, radio listening, and internet usage do not show significant associations with depression status, TV viewing does. The findings show that those who read newspapers less than once a week reported 2.56% cases of depression while those that read newspapers at least once a week reported 2.53% cases of depression. However, there is no significant association between magazine reading frequency and depression status. Almost similar results are reported for listening to radio and using of internet. It is shown from the findings that 3.05% of those who watch TV less than one day a week reported cases of depression while 2.18% was reported in those that watch TV at least one day a week. The findings are indicative of a marginal but statistically significant correlation between TV watching frequency and depression status, suggesting that those with depression are more likely not to watch TV.

Socioeconomic factors such as wealth index and occupation, demonstrate significant correlations with the incidence of reporting positive for depression or anxiety. This suggests that socioeconomic status may be linked to depression in the general population. In particular, in terms of wealth index, depression was significantly higher middle-income individuals at 2.76%. A wide range of occupations is represented, with significant variance in reported depression. The highest rates of depression are seen in agriculture employees (3.62%) a professional/technical/managerial roles (2.97%). Those not working report depression at a rate of 1.41%. The results indicate a statistically significant difference in the rates of depression across different occupations.

The study also sought to establish the influence of health conditions on depression. From the findings, the vast majority of the population does not have hypertension. A significantly lower percentage (9.08%) of those who reported depression or anxiety are hypertensive. The results also show that there is a strong statistical association between depression and hypertension, suggesting that depression is more prevalent among those with hypertension. A very high percentage of the population does not report both depression and diabetes (97.5%) while 6.01% of those who reported to have been diagnosed with depression are diabetics. In addition, the results suggest that there is a significant association between depression and diabetes, with depression being more common among those with diabetes. The proportion of heart disease is very low in the general population, with 8.88% reporting both depression and heart disease. Furthermore, depression is reported in 9.63% of those having lung disease.

### Country distribution of self-reported depression

**Fig. 1:**
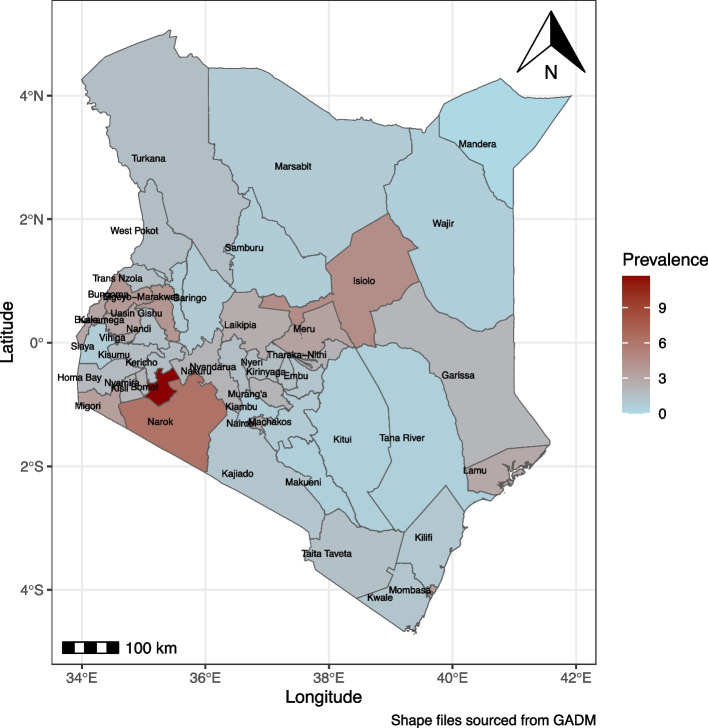
The Country depression prevalence profile

Depression remains a significant health concern across Kenya, with marked variation in self-reported prevalence rates across different counties. As illustrated in Fig. 1, Bomet County has the highest reported rate of depression, at 11.8%, indicating a substantial mental health burden in this region. Narok County follows, with a depression proportion of 6.05%, while Isiolo County reports a rate of 5.36%. On the opposite end of the spectrum, Mandera County records the lowest proportion of self-reported depression, closely followed by Wajir, Marsabit, Tana River, and Kitui Counties, which also exhibit relatively low rates. Urban counties, including Nairobi, Mombasa, Kisumu, and Nakuru, show moderate levels of self-reported depression, reflecting the mixed urban-rural distribution and possibly diverse socio-economic factors impacting mental health in these areas.

### Factors associated with depression

From the findings in Table 2 residing in an urban area (compared to rural) is associated with a 3% increase in the odds of reporting depression, and this effect is not statistically significant $(p = 0.39> 0.05)$ after adjusting for the residence. The results show that females are at a higher likelihood of reporting depression as opposed to males (aOR: 1.30, 95% CI 1.23-1.38). Older individuals aged 50-54 years are highly likely to report depression or anxiety (aOR: 3.64, 95%CI: 3.13-4.23) relative to those aged 15-19 years. Whereas education level and religion were factors associated with depression, having lower level of education decreased the odds of reporting depression or anxiety, for example, those with higher education had aOR: 2.48, (95%CI:2.14- 2.89) higher likelihood of reporting depression or anxiety than those with no education. In the religion category, being an atheist (having no religion) increased the odds of reporting depression or anxiety (aOR: 1.67, 95%CI:1.48-1.89) than being in the Catholic church. Ethnicity was also established to be significantly associated with depression. However, being a Maasai, Luhyia or Meru poses a high likelihood of reporting depression or anxiety (aOR: 8.97, 95%CI:6.12 - 13.15, aOR: 4.44, 95%CI:3.09-6.38 and aOR: 4.04, 95%CI:2.79-5.85, respectively) as compared to being Embu. Those who do watch television at least one day a week are at lower likelihood of reporting depression or anxiety (aOR: 0.68, 95%CI:0.65-0.72) as opposed to those who do watch TV less one day a week. In terms of wealth and occupation, whereas wealth index and occupation were factors associated with depression, middle income earners are significantly at high likelihood of reporting depression (aOR: 1.12, 95%CI:1.03 - 1.22) compared to poorest individuals. Hypertension, diabetes, heart disease, and lung disease predicted hihger likelihood of reporting depression. Being hypertensive, diabetic, and having heart and lung diseases increased the odds of reporting depression by at least two-fold, (aOR:3.71, 95% CI: 3.49-3.95, aOR:1.14, 95% CI: 0.96-1.35, aOR:3.10, 95% CI: 2.68-3.57 and aOR:3.21, 95% CI: 2.84-3.62, respectively).

**Table 2:** Univariable and multivariable level analysis of factors associated with self-reported depression among people aged 15-54 years in Kenya using survey-weighted logistic regression

Characteristics	Univariate		Multivariable	
	OR (95%CI)	*P*-value	aOR (95%CI)	*P*-value
Age				
15-19	1.00		1.00	
20-24	1.68(1.51-1.88)	<0.0001	1.32(1.18-1.48)	<0.0001
25-29	2.80(2.53-3.10)	<0.0001	2.13(1.91-2.37)	<0.0001
30-34	3.64(3.29-4.03)	<0.0001	2.69(2.41-3.00)	<0.0001
35-39	4.72(4.27-5.21)	<0.0001	3.35(3.00-3.73)	<0.0001
40-44	5.83(5.28-6.45)	<0.0001	3.84(3.44-4.29)	<0.0001
45-49	4.31(3.87-4.81)	<0.0001	2.70(2.40-3.03)	<0.0001
50-54	5.23(4.54-6.01)	<0.0001	3.64(3.13-4.23)	<0.0001
Sex				
Male	1.00		1.00	
Female	1.31(1.25-1.37)	<0.0001	1.30(1.23-1.38)	<0.0001
Residence				
Urban	1.00		1.00	
Rural	1.14(1.08-1.20)	<0.0001	1.03(0.96-1.11)	0.39
Education level				
No education	1.00		1.00	
Primary	1.15(1.02-1.30)	0.024	1.58(1.39-1.80)	<0.0001
Secondary	0.77(0.68-0.87)	<0.0001	1.67(1.45-1.91)	<0.0001
Higher education	1.16(1.02-1.32)	0.020	2.48(2.14-2.89)	<0.0001
Religion				
Catholic	1.00		1.00	
Protestant	0.84(0.79-0.89)	<0.0001	0.80(0.75-0.86)	<0.0001
Evangelical	0.92(0.86-0.99)	0.018	0.86(0.80-0.93)	<0.0001
African Instituted Churches	0.83(0.75-0.91)	<0.0001	0.79(0.72-0.87)	<0.0001
Orthodox	1.28(0.85-1.94)	0.240	1.48(0.97-2.25)	<0.0001
Islam	0.85(0.76-0.94)	0.002	1.35(1.19-1.54)	<0.0001
Hindu	0.63(0.36-1.10)	0.102	0.44(0.25-0.79)	0.006
Tradition	0.51(0.26-1.02)	0.056	0.62(0.31-1.24)	0.177
No religion	1.62(1.44-1.82)	<0.0001	1.67(1.48-1.89)	<0.0001
Other	0.74(0.63-0.88)	0.001	0.85(0.71-1.01)	0.06
Ethnicity				
Embu	1.00		1.00	
Kalenjin	3.44(2.40-4.93)	<0.0001	3.89(2.70-5.59)	<0.0001
Kamba	2.25(1.57-3.24)	<0.0001	2.73(1.89-3.95)	<0.0001
Kikuyu	2.88(2.01-4.12)	<0.0001	3.23(2.24-4.64)	<0.0001
Kisii	3.25(2.25-4.70)	<0.0001	4.04(2.79-5.85)	<0.0001
Luhya	3.59(2.51-5.14)	<0.0001	4.44(3.09-6.38)	<0.0001
Luo	2.94(2.05-4.21)	<0.0001	3.48(2.42-5.02)	<0.0001
Maasai	5.40(3.70-7.87)	<0.0001	8.97(6.12-13.15)	<0.0001
Meru	4.11(2.85-5.92)	<0.0001	4.09(2.83-5.92)	<0.0001
Mijikenda	2.55(1.76-3.70)	<0.0001	2.78(1.90-4.08)	<0.0001
Somali	1.86(1.24-2.79)	0.003	2.21(1.44-3.39)	<0.0001
Taita	2.58(1.65-4.03)	<0.0001	2.59(1.65-4.08)	<0.0001
Other	1.72(1.17-2.55)	0.006	2.27(1.53-3.37)	<0.0001
Media exposure				
Newspaper/magazine				
Less than one day a week	1.00		1.00	
At least one day a week	0.98(0.92-1.06)	0.654	1.03(0.95-1.11)	0.504
Radio				
Less than one day a week	1.00		1.00	
At least one day a week	0.80(0.76-0.84)	<0.0001	0.79(0.75-0.83)	<0.0001
Television				
Less than one day a week	1.00		1.00	
At least one day a week	0.71(0.68-0.74)	<0.0001	0.68(0.65-0.72)	<0.0001
Internet				
Less than one day a week	1.00		1.00	
At least one day a week	0.86(0.82-0.90)	<0.0001	0.90(0.84-0.96)	0.001
Wealth Index				
Poorest	1.00		1.00	
Poorer	0.97(0.90-1.05)	0.49	1.00(0.92-1.09)	0.953
Middle	1.03(0.95-1.11)	0.486	1.12(1.03-1.22)	0.010
Richer	0.93(0.86-1.01)	0.082	1.10(1.00-1.22)	0.051
Richest	0.87(0.80-0.94)	0.001	0.95(0.84-1.08)	0.457
Has hypertension				
No	1		1.00	
Yes	4.96(4.68-5.25)	<0.0001	3.71(3.49-3.95)	<0.0001
Has diabetes				
No	1		1.00	
Yes	2.64(2.25-3.10)	<0.0001	1.14(0.96-1.35)	0.148
Has heart diseases				
No	1		1.00	
Yes	3.91(3.42-4.46)	<0.0001	3.10(2.68-3.57)	<0.0001
Has lung disease				
No	1		1.00	
Yes	4.32(3.84-4.86)	<0.0001	3.21(2.84-3.62)	<0.0001
Occupation				
Not working	1		1.00	
Professional/Technical/Managerial	2.14(1.98-2.31)	<0.0001	1.34(1.23-1.47)	<0.0001
Clerical	1.76(1.40-2.22)	<0.0001	1.15(0.90-1.45)	0.262
Sales	1.79(1.61-2.00)	<0.0001	1.19(1.06-1.33)	0.004
Agriculture - self employed	1.72(1.35-2.18)	<0.0001	1.15(0.90-1.47)	0.275
Agriculture - employee	2.51(2.34-2.70)	<0.0001	1.81(1.67-1.96)	<0.0001
Household and domestic	2.45(2.21-2.72)	<0.0001	1.78(1.59-1.98)	<0.0001
Services	2.05(1.84-2.29)	<0.0001	1.52(1.35-1.71)	<0.0001
Skilled manual	2.07(1.89-2.26)	<0.0001	1.91(1.72-2.11)	<0.0001
Unskilled manual	2.09(1.92-2.29)	<0.0001	1.49(1.36-1.64)	<0.0001

## Discussions

This paper presents a comprehensive investigation into the socio-economic, demographic and health-related factors associated with self-reported depression in Kenya using data from the Demographic and Health Survey of the year 2022. Our findings indicate that the prevalence of self reported depression varies across different demographic groups. It varies according to age, gender, residence, education level, religion, and ethnicity, with each factor demonstrating statistically significant differences in the rates of reported depression. These variations may be due to economic inequalities, fast-paced lifestyles, and differing societal expectations between urban and rural settings. The results also provide insight into media consumption and socio-economic variables in relation to depression. The multivariable analysis reveals significant associations between various factors and the odds of depression, shedding light on crucial determinants that should be considered in mental health interventions. The study encompassed a diverse range of demographic, socio-economic, and health-related variables, contributing valuable insights into the multifaceted nature of depression.

The proportion of self-reported depression in Kenya was found to be 2.6%, indicating that a notable segment of the study participants had been diagnosed with depression or anxiety. This finding emphasizes the potential burden of depressive disorders in the population, reflecting the specific questions asked in the study related to mental health diagnoses. This finding is comparable to the WHO report, which estimated that 3.8% of the global population experiences depression [25]. However, it is important to note that the WHO data represent global estimates across various populations, which may differ in demographic, cultural, and socioeconomic characteristics compared to the specific population studied in Kenya. Differences between the reported rates may be influenced by these population variations, as well as by the definitions and methods used to measure depression. For example, the WHO estimate encompasses a broader context, potentially including diverse diagnostic criteria and self-reporting methods across countries. In contrast, our study’s findings are based on specific questions about diagnosed depression or anxiety among our sample population, which could influence the comparability of the rates.

According to our findings, the proportion of self-reported depression in Kenya is lower than that reported in Nigeria, which stands at 3.9% [26]. Although not statistically significant, residing in an urban area showed a 3% increase in the odds of self-reported depression, emphasizing the relationship with residency of individuals. Females were found to be at a higher likelihood of reporting depression compared to males, aligning with existing literature on gender disparities in mental health [19–21, 27]. This finding is also consistent with the findings in [26] in which it is reported that even though burden of depression varies by WHO regions, it ranges from 2.6% in males located within the Pacific region to 5.9% in females located within Africa region. Psychologically, the female gender is prone to repetitive thinking with higher expectations from society than males which may be attributed to the higher proportion of depression in females than males in the population.

Age played a substantial role, with individuals aged 50-54 years having a significantly higher likelihood of reporting symptoms of depression or anxiety. This aligns with prior research linking age with increased vulnerability to mental health challenges as individuals age [28, 29]. Education level was inversely associated with self-reported depression, where individuals with lower educational attainment reported lower odds of depression, suggesting that awareness and recognition of mental health issues may be higher among more educated individuals. Religion and ethnicity were significant factors; atheists and members of specific ethnic groups exhibited higher odds of self-reporting depression, reflecting the role of cultural and religious factors in mental health [30, 31]. The lack of social support commonly available within religious groups may partly explain higher depression rates among atheists. Although magazine readership, radio listening, and internet use were not significantly associated with depression, regular television viewing was linked to lower odds of self-reported depression, suggesting potential positive effects of media engagement on mental health [19]. Socioeconomic factors, particularly wealth index and occupation, showed strong associations with mental health outcomes; middle-income individuals reported a higher likelihood of depression, underscoring the relevance of economic stability in mental health [32]. Chronic health conditions, including hypertension, diabetes, heart disease, and lung disease, were significant predictors of depression risk, reinforcing the interconnected nature of mental and physical health and the need for integrated healthcare approaches [33].

Our findings indicate that there is a strong link between hypertension, diabetes, heart disease, lung disease and depression consistent with results in [34–36]. There is always a general concurrence by both patients and healthcare professionals that chronic conditions frequently trigger feelings of grief and hopelessness [37, 38]. Individuals diagnosed with a chronic ailment encounter a myriad of losses and in most instance they must relinquish certain activities that once brought them joy or solace, expend additional effort on tasks that were previously effortless, and grapple with potential limitations in their physical abilities, abruptly jeopardizing significant aspects of their future, thus depression crippling in [38]. Furthermore, our findings add to emerging research which has unveiled a bidirectional relationship between physical health conditions and mental health, particularly depression [39, 40]. Individuals grappling with chronic diseases like hypertension, diabetes, heart disease, or lung disorders often face increased susceptibility to depression. Conversely, individuals with depression may exhibit higher instances of these physical ailments due to shared risk factors, including unhealthy lifestyle habits, chronic stress, inflammation, and altered immune function [34, 35].

In light of our findings, we recommend further research into the specific psychological issues facing communities to inform the development of targeted interventions. Such research could lay the groundwork for future community-based rehabilitation or counseling services aimed at addressing the mental health needs of these populations. Additionally, expanding access to mental health care, including counseling and other psychosocial supports, should be prioritized to improve the quality of life for individuals affected by depression. Monitoring the population’s mental health regularly can also offer insights into trends and help identify high-risk groups. Preventing and managing depression effectively has broader benefits, potentially reducing the burden on the healthcare system and enhancing overall public health outcomes.

The study also recommends that given the intricate comorbidity of chronic diseases (hypertension, diabetes, heart disease, or lung disorders) underscores the importance of adopting a holistic approach to healthcare. Addressing not only the physical symptoms but also the psychological well-being is crucial in order to avert depression. Comprehensive care strategies that integrate both physical and mental health components are pivotal for effective management and prevention. Collaborative efforts among healthcare professionals across various disciplines are essential to provide holistic care, offer support, and enhance the overall well-being of individuals affected by this complex interplay of these health conditions. While there may be a correlation between media access and mental health awareness, recommending television as a preventative measure against depression requires further evidence, likely through hypothesis-driven studies focusing on media’s role in mental health awareness or community support. In our study, we included ethnicity in the multivariable analysis to reflect the diverse socio-cultural context in Kenya, which can influence health behaviours and access to mental health resources. Thus, ethnicity is included to better understand how socio-cultural differences might intersect with mental health outcomes to ensure that public health interventions are more inclusive and relevant across different demographic groups.

Based on the assessment approach used in the KDHS, we recommend that future surveys incorporate a more detailed, validated tool such as the Patient Health Questionnaire (PHQ), which is widely recognized for effectively measuring depressive symptoms across diverse populations, including adolescents. The DHS depression and anxiety module, currently utilized, includes general self-report questions that may not capture the refined clinical aspects of depression and anxiety, particularly among teenagers aged 15 to 19. Using a clinically validated tool like the PHQ could enhance the accuracy and reliability of mental health prevalence estimates and provide a more comprehensive understanding of mental health challenges within specific age groups. This change would improve the quality of mental health data collected, allowing policymakers and health practitioners to better address population-specific needs in mental health interventions and resource allocation.

### Strengths and limitations

The major strength of our study lies in the use of survey-weighted logistic regression, a robust statistical technique well-suited for handling complex DHS data. This approach ensures that estimates are representative of the population, reliable, and account for the survey design, thereby enhancing the validity of the findings and supporting informed decision-making. Additionally, the study’s use of a nationally representative DHS dataset allows for broad generalizability of results within Kenya hence providing valuable insights into mental health at the population level.

The present study contributes to understanding the mental health landscape in Kenya but acknowledges certain limitations. It relies on self-reported, cross-sectional data, which limits the capacity to establish cause-and-effect relationships between depression and the variables under study. Furthermore, while the survey gathered data on anxiety and depression, it did not delve into the specific factors contributing to these conditions, thus lacking in-depth insights into the contextual and situational elements that may drive mental health issues. Additionally, the survey did not capture details on the severity of socio-economic factors or chronic diseases associated with depression, potentially overlooking the differential impacts of these factors on mental health. This gap highlights a need for further investigation into these relationships.

Moreover, the 2022 KDHS assessed self-reported diagnoses of depression, likely resulting in an underestimation of prevalence compared to the outcomes from using a more detailed assessment tool such as the Patient Health Questionnaire (PHQ-2 or PHQ-9). Despite the absence of PHQ-2 or PHQ-9 measures, this study offers valuable insights into the burden of depression in Kenya, establishing a foundation for future research and interventions that address mental health challenges within the population.

## Conclusion

This study emphasizes the importance of a comprehensive approach to understanding mental health issues in Kenya. Our investigation of various social, economic, and psychological factors associated with anxiety and depression provides a significant foundation for targeted interventions. The findings emphasize the need to consider these determinants in shaping effective strategies for both preventing and managing depression within the population. By addressing these factors collectively, stakeholders can support more meticulous and impactful mental health strategies tailored to the Kenyan context.

## Data Availability

Data are available in a public, open access repository. The data set supporting the conclusions of this article is available in the DHS Programme available at https://dhsprogram.com/.

## Abbreviations

DHS: Demographic and Health Surveys
KDHS: Kenya Demographic and Health Survey
HIV: Human Immunodeficiency Virus
PHQ: Patient Health Questionnaire
PHQ-2: Patient Health Questionnaire - 2 (a shorter version)
PHQ-9: Patient Health Questionnaire - 9 (a more detailed version)
GIS: Geographic Information System
KNBS: Kenya National Bureau of Statistics
ICF: Inner City Fund
STATA: A statistical software package (often used in research)
R: A programming language and software environment for statistical computing
R Studio: An integrated development environment (IDE) for R
OR: Odds Ratio
CI: Confidence Interval
aOR: Adjusted Odds Ratio
WHO: World Health Organization
EA: Enumeration Area
GADM: Database of Global Administrative Areas (used for mapping)
